# Mutation spectrum data for *Saccharomyces cerevisiae psf1-1 pol2-M644G* mutants

**DOI:** 10.1016/j.dib.2022.108223

**Published:** 2022-04-29

**Authors:** Michal Dmowski, Karolina Makiela-Dzbenska, Malgorzata Jedrychowska, Milena Denkiewicz-Kruk, Iwona J. Fijalkowska

**Affiliations:** Institute of Biochemistry and Biophysics, Polish Academy of Sciences, Pawinskiego 5A, 02-106 Warsaw, Poland

**Keywords:** DNA polymerase epsilon (Pol ε), CMG (Cdc45 Mcm2-7 GINS), Replication fork, DNA replication fidelity, Genome stability

## Abstract

DNA replication in *Saccharomyces cerevisiae* and other eukaryotes is performed mainly by polymerase epsilon (Pol ε) on the leading strand and polymerase delta (Pol δ) on the lagging strand. Using a mutant form of a DNA polymerase enables tracking its signature in the replicated DNA. Here, we used the *pol2-M644G* allele encoding the catalytic subunit of Pol ε to analyse its contribution to DNA replication in yeast with the *psf1-1* allele of an essential gene encoding a subunit of the GINS complex. GINS is involved in the recruitment of Pol ε, the major leading strand replicase. Thus, its defective functioning can affect the involvement of Pol ε in DNA replication. Together with Cdc45 and Mcm2-7, GINS forms the CMG helicase complex. Our DNA sequencing data enable the observation of changes in the mutational spectra in the *URA3* reporter gene cloned in two orientations regarding the nearest ARS. The data presented in this article support the study "Increased contribution of DNA polymerase delta to the leading strand replication in yeast with an impaired CMG helicase complex” [1].

## Specifications Table

**Table:** 

Subject	*Genetics: General*
Specific subject area	*DNA replication in eukaryotes*
Type of data	*Table* *Figure*
How the data were acquired	*Selection of mutant clones, DNA sequencing*
Data format	*Raw* *Analysed*
Description of data collection	*Yeast cells with pol2-M644G or psf1-1 pol2-M644G mutations in the rev3Δ msh6Δ background were cultured at 23* °*C (until they reached the stationary phase) and plated on 5′-Fluoroorotic acid (5′-FOA)-containing media to select clones with mutations within the reporter URA3 gene. The URA3 sequence was then amplified and sequenced. Mutation rates were calculated as described previously*
Data source location	*Institute of Biochemistry and Biophysics, Polish Academy of Sciences, Warsaw, Poland*
Data accessibility	*With the article. Raw data can be found at Mendeley Data* (doi:10.17632/m5pw82p9p5.1)*.*
Related research article	M. Dmowski, M. Jedrychowska, K. Makiela-Dzbenska, M. Denkiewicz-kruk, S. Sharma, A. Chabes, H. Araki, I.J. Fijalkowska, Increased contribution of DNA polymerase delta to the leading strand replication in yeast with an impaired CMG helicase complex, DNA Repair (Amst). 110 (2022) 103,272. https://doi.org/10.1016/j.dnarep.2022.103272

## Value of the Data

• These are the first data on mutation specificity of the M644G-Pol2 variant alone and combined with the *psf1-1* allele under conditions of polymerase zeta (Pol ζ) inactivation (*REV3* deletion) and mismatch-repair mechanism deficiency (*MSH6* deletion).
• These data can be beneficial for researchers deciphering DNA replication mechanisms and studying the fidelity of this process as well as the proteins involved in it.
• These data provide information on the mutation spectra of the *pol2-M644G* in *psf1-1* mutant cells.

## 1 Data Description

**Fig. 1:**
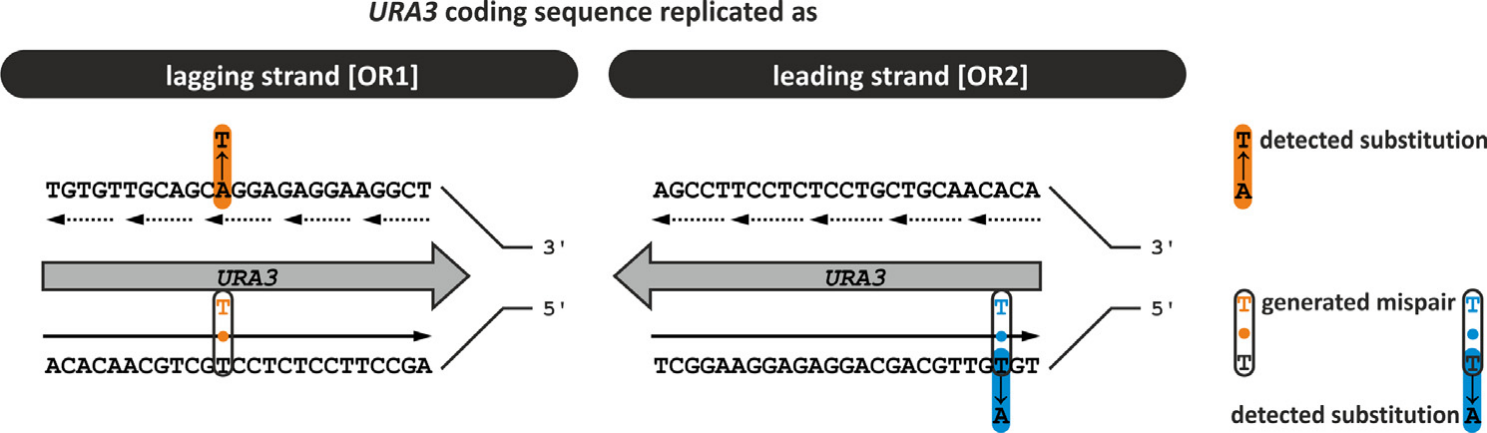
M644G-Pol ε signature results from T•T mispairs generated during leading strand replication. *URA3* reporter gene is cloned in two orientations (OR1 and OR2) in the vicinity of ARS306. Its coding sequence is replicated as the lagging strand in OR1 and as the leading strand in OR2. Therefore, T•T mispairs can be detected as A to T substitutions in *URA3* OR1 and T to A in *URA3* OR2.

DNA replication in eukaryotic cells is conducted primarily by Pol ε and Pol δ on the leading and lagging strand, respectively. This division of labour may be changed under some conditions e.g. when the replication process is perturbed. Using specific mutants in genes encoding catalytic subunits of DNA polymerases, one can track the involvement of either replicase in DNA synthesis. The M644G variant of Pol2, the catalytic subunit of Pol ε demonstrates significantly higher rates of T•dT mispairs than A•dA [2] (Fig. 1). Here, the signature of *pol2-M644G* was analysed in the *psf1-1* mutant in the *rev3Δ msh6Δ* background. *PSF1* encodes the psf1 essential subunit of the GINS complex, a component of the CMG helicase [3]. The *psf1-1* subunit demonstrates impaired interaction with another GINS subunit - Psf3 [1,4], resulting in impaired functioning of the complex, what can affect the contribution of Pol ε to DNA replication. Deletion of *REV3* inactivates DNA polymerase zeta (Pol ζ) activity partially responsible for the increased mutation rates in the *psf1-1* mutant [4]. The *MSH6* gene was inactivated to impair the mismatch repair mechanism (MMR), correcting replication errors. The mutation spectra were analysed in the reporter gene *URA3* cloned in two orientations (OR1 and OR2) close to ARS306. Obtained mutation rates and spectra are shown in Table 1. Raw data associated with these results are accessible in a file deposited at Mendeley Data (doi:10.17632/m5pw82p9p5.1). It includes positions as well as the sequence context of mutated nucleotides.

**Table 1:** Mutation rates calculated for specific mutation types in the *URA3* sequence. Data for control strains OR1, OR2, *psf1-1* OR1, and *psf1-1* OR2 are presented in the related research article [1].

Type	*pol2-M644G* OR1		*pol2-M644G* OR2		*psf1-1 pol2-M644G* OR1		*psf1-1 pol2-M644G* OR2	
**Transitions**	83^a^	**38.54**^b^	68	**44.70**	160	**62.81**	228	**101.07**

T→C	6	**2.79**	17	**11.17**	25	**9.81**	61	**27.04**
A→G	2	**0.93**	3	**1.97**	6	**2.36**	6	**2.66**
C→T	20	**9.29**	17	**11.17**	20	**7.85**	39	**17.29**
G→A	55	**25.54**	31	**20.38**	109	**42.79**	122	**54.08**

**Transversions**	69	**32.04**	35	**23.01**	138	**54.17**	108	**47.87**

G→T	36	**16.72**	23	**15.12**	93	**36.51**	42	**18.62**
C→A	2	**0.93**	2	**1.31**	4	**1.57**	19	**8.42**
T→G	2	**0.93**	3	**1.97**	3	**1.18**	16	**7.09**
A→C	0	**0.00**	0	**0.00**	4	**1.57**	7	**3.10**
A→T	26	**12.07**	0	**0.00**	26	**10.21**	2	**0.89**
*A→T at 686*^c^	6	**2.79**	0	**0.00**	5	**1.96**	1	**0.44**
*A→T at OS*^d^	20	**9.29**	0	**0.00**	21	**8.24**	1	**0.44**
T→A	3	**1.39**	7	**4.60**	6	**2.36**	21	**9.31**
G→C	0	**0.00**	0	**0.00**	1	**0.39**	1	**0.44**
C→G	0	**0.00**	0	**0.00**	1	**0.39**	0	**0.00**

**Indels**	2	**0.93**	0	**0.00**	8	**3.14**	2	**0.89**

single deletions	1	**0.46**	0	**0.00**	4	**1.57**	1	**0.44**
≥2 deletions	0	**0.00**	0	**0.00**	0	**0.00**	0	**0.00**
single insertions	1	**0.46**	0	**0.00**	4	**1.57**	1	**0.44**
≥2 insertions	0	0.00	0	**0.00**	0	**0.00**	0	**0.00**

**TOTAL**	154	**71.51**	103	**67.70**	306	**120.12**	338	**149.83**

**95% CI**		**59.4** **83.3**		**52.3** **80.9**		**90.5** **153.6**		**140.4** **207.7**

a Number of events identified for given classes.

b Mutation rates [5-FOA^R^ × 10^−6^] for specific mutation types are shown in boldface.

c Specific hotspot positions in the *URA3* coding sequence are indicated.

d OS – Other Sites.

## 2 Experimental Design, Materials and Methods

*S. cerevisiae* strains used for mutation spectra analysis are listed in Table 2. They were derivatives of SNM70, and SNM79 [5] strains kindly provided by T. A. Kunkel (NIEHS, USA). These strains contained the reporter gene *URA3* cloned in two orientations (OR1 and OR2) in respect to the nearest origin of replication (ARS306). SNM70 and SNM79 strains contained the *pol2-M644G* allele additionally. *NAT1* and *HPH* genes were used to inactivate *REV3* and *MSH6* genes, respectively. To do this, primers pairs Rev3_UPTEF (CAATACAAAACTACAAGTTGTGGCGAAATAAAATGTTTGGAAATGAGATCTGTTTAGCTTGCC) - Rev3_DNTEF (ATAACTACTCATCATTTTGCGAGACATATCTGTGTCTAGATTATTCGAGCTCGTTTTCGACAC) and msh6UTEF (CAGATAAGATTTTTTAATTGGAGCAACTAGTTAATTTTGACAAAGCCAATTTGAACTCCAAAAGATCTGTTTAGCTTGCC) - msh6DTEF (CAACGACCAAAACTTTAAAAAAAATAAGTAAAAATCTTACATACATCGTAAATGAAAATATTCGAGCTCG TTTTCGACAC) were used with pAG25 and pAG32 plasmids as template, respectively. Yeast transformation was done using the LiAc/ssDNA/PEG method [6]. Chromosomal DNA from yeast was purified using the Genomic Mini AX Yeast Spin Kit (A&A Biotechnology, Gdansk, Poland). The presence of the *rev3*Δ::*NAT1* cassette in nourseothricin-resistant transformants was verified by PCR with primers Rev3-R4 (TGACCACTCACATGGCGCTTTG) – Rev3A (AATTCTGCCAATCTATTTGATCTTG) – nat1UO (ACCGGTAAGCCGTGTCGTCAAG) and Rev3-F4 (AAAGGGCGAGCACAACTACTAC) – Rev3D (CACCAGATAGAGTTTTGAACGAAAT) – nat1DO (GCTTCGTGGTCGTCTCGTACTC). The presence of the *msh6*Δ::*HPH* cassette in hygromycin-resistant transformants was verified by multiplex PCR with primers MSH6-UO (TAAAGTCGCTGGAGTAGG) – msh6up2 (GAATCCTTGGAGGAAGAC) – HPH-UO (ACAGACGTCGCGGTGAGTTCAG) and MSH6-DO (TCAAGCACCATCCTCAAG) – msh6dw2 (CCCATTCTTGCCCAAGATGC) – HPH-DO (TCGCCGATAGTGGAAACCGACG). The *PSF1-LEU2* and *psf1-1-LEU2* alleles were introduced into yeast strains as described previously [4]. Additionally, *pol2-M644G rev3*Δ *psf1-1 msh6*Δ strains were obtained by tetrad dissection from heterozygous diploid strains.

**Table 2:** Yeast strains used in this study.

Strain	Relevant genotype	Description	Source
**SNM70**	*pol2M644G agp1::URA3-OR1*		[5]
**Y471**	*pol2M644G agp1::URA3-OR1 rev3Δ*	*REV3* disruption in SNM70	This work
**Y475-1**	*pol2M644G agp1::URA3-OR1 rev3Δ PSF1*	*PSF1-LEU2* derivative of Y471	This work
**Y475-2**	*pol2M644G agp1::URA3-OR1 rev3Δ PSF1*	*PSF1-LEU2* derivative of Y471	This work
**Y487-2**	*pol2M644G agp1::URA3-OR1 rev3Δ PSF1 msh6Δ*	*MSH6* disruption in Y475*-*1	This work
**Y488-1**	*pol2M644G agp1::URA3-OR1 rev3Δ PSF1 msh6Δ*	*MSH6* disruption in Y475*-*2	This work
**Y483-1**	*pol2M644G agp1::URA3-OR1 rev3Δ psf1-1*	*psf1-1-LEU2* derivative of Y471	This work
**Y483-2**	*pol2M644G agp1::URA3-OR1 rev3Δ psf1-1*	*psf1-1-LEU2* derivative of Y471	This work
**Y483-3**	*pol2M644G agp1::URA3-OR1 rev3Δ psf1-1*	*psf1-1-LEU2* derivative of Y471	This work
**Y508-1**	*pol2M644G agp1::URA3-OR1 rev3Δ psf1-1 msh6Δ*	*MSH6* disruption in Y481*-*1	This work
**Y509-3**	*pol2M644G agp1::URA3-OR1 rev3Δ psf1-1 msh6Δ*	*MSH6* disruption in Y481*-*2	This work
**Y510-8**	*pol2M644G agp1::URA3-OR1 rev3Δ psf1-1 msh6Δ*	*MSH6* disruption in Y481*-*3	This work
**Y640-A**	*POL2/pol2M644G agp1::URA3-OR1/agp1::URA3-OR1 rev3Δ /rev3Δ PSF1/psf1-1 MSH6/msh6Δ*	Diploid strain	This work
**Y640-B**	*POL2/pol2M644G agp1::URA3-OR1/agp1::URA3-OR1 rev3Δ /rev3Δ PSF1/psf1-1 MSH6/msh6Δ*	Diploid strain	This work
**Y641**	*pol2M644G agp1::URA3-OR1 rev3Δ psf1-1 msh6Δ*	Segregant of Y640-A	This work
**Y643**	*pol2M644G agp1::URA3-OR1 rev3Δ psf1-1 msh6Δ*	Segregant of Y640-B	This work
**SNM79**	*pol2M644G agp1::URA3-OR2*		[5]
**Y472**	*pol2M644G agp1::URA3-OR2 rev3Δ*	*REV3* disruption in SNM79	This work
**Y476-1**	*pol2M644G agp1::URA3-OR2 rev3Δ PSF1*	*PSF1-LEU2* derivative of Y472	This work
**Y476-2**	*pol2M644G agp1::URA3-OR2 rev3Δ PSF1*	*PSF1-LEU2* derivative of Y472	This work
**Y489-2**	*pol2M644G agp1::URA3-OR2 rev3Δ PSF1 msh6Δ*	*MSH6* disruption in Y476*-*1	This work
**Y490-1**	*pol2M644G agp1::URA3-OR2 rev3Δ PSF1 msh6Δ*	*MSH6* disruption in Y476*-*2	This work
**Y484-1**	*pol2M644G agp1::URA3-OR2 rev3Δ psf1-1*	*psf1-1-LEU2* derivative of Y472	This work
**Y484-2**	*pol2M644G agp1::URA3-OR2 rev3Δ psf1-1*	*psf1-1-LEU2* derivative of Y472	This work
**Y484-3**	*pol2M644G agp1::URA3-OR2 rev3Δ psf1-1*	*psf1-1-LEU2* derivative of Y472	This work
**Y511-6**	*pol2M644G agp1::URA3-OR2 rev3Δ psf1-1 msh6Δ*	*MSH6* disruption in Y484*-*1	This work
**Y512-2**	*pol2M644G agp1::URA3-OR2 rev3Δ psf1-1 msh6Δ*	*MSH6* disruption in Y484*-*2	This work
**Y513-2**	*pol2M644G agp1::URA3-OR2 rev3Δ psf1-1 msh6Δ*	*MSH6* disruption in Y484*-*3	This work
**Y640-1**	*POL2/pol2M644G agp1::URA3-OR2/agp1::URA3-OR2 rev3Δ /rev3Δ PSF1/psf1-1 MSH6/msh6Δ*	Diploid strain	This work
**Y640-D**	*POL2/pol2M644G agp1::URA3-OR2/agp1::URA3-OR2 rev3Δ /rev3Δ PSF1/psf1-1 MSH6/msh6Δ*	Diploid strain	This work
**Y646**	*pol2M644G agp1::URA3-OR2 rev3Δ psf1-1 msh6Δ*	Segregant of Y640*-*1	This work
**Y647**	*pol2M644G agp1::URA3-OR2 rev3Δ psf1-1 msh6Δ*	Segregant of Y640*-*1	This work
**Y648**	*pol2M644G agp1::URA3-OR2 rev3Δ psf1-1 msh6Δ*	Segregant of Y640-D	This work

For antibiotic resistance selection, yeast were grown at 23 °C on YPD (1% Bacto-yeast extract, 2% Bacto-peptone, 2% glucose solidified with 2% Bacto-agar) supplemented with hygromycin B 300 µg/ml (Bioshop, Burlington, Canada) or nourseothricin 100 µg/ml (Werner BioAgents, Jena, Germany). For mutation rate and spectra analyses, yeast were grown at 23 °C on SD medium (0.67% yeast nitrogen base without amino acids, 2% glucose) supplemented with appropriate amino acids and nitrogenous bases. SD medium solidified with 2% Bacto-agar with the addition of 1 mg/ml 5-fluoroorotic acid (5-FOA) (US Biological, Salem, MA, USA) was used for *URA3* mutants selection [7].

For mutation rate analyses, each of two or three independent isolates of each strain was used to inoculate at least eight cultures (2 ml each) grown at 23 °C until stationary phase. Then, appropriate dilutions of yeast cultures were plated on nonselective and selective (supplemented with 5-FOA for selection of *URA3* mutants) media. After 4–7 days, yeast colonies were counted. To calculate spontaneous mutation rates, the *µ = ƒ/ln(Nµ)* equation was used [*µ* - mutation rate per round of DNA replication; *ƒ* - mutant frequency (cell count from selective media divided by the cell count from nonselective media), and *N* - total population] [8]. Median values of mutation rates and 95% confidence intervals were calculated (GraphPad Prism software).

To characterize the mutation spectrum in the *URA3* reporter gene, 103-338 5-FOA-resistant colonies were analyzed for each strain. Each colony represents an independent culture that was diluted and plated on a 5-FOA-containing SD medium. Primers URA3F393 (AACGAAGGAAGGAGCACAGAC) and URA3R412 (CCGAAATTCCTGGGTAATAAC) were used to PCR-amplify the *URA3* gene from 5-FOA resistant clones and for sequencing of the PCR product. The contribution of either mutation type to overall mutagenesis was calculated by dividing the number of specific events by the total number of mutations. Specific mutation rates were calculated proportionally to their contribution to the mutation spectrum.

## Ethics Statements

This work involved neither human subjects, nor animal experiments, and adheres to Ethics in publishing standards.

## CRediT authorship contribution statement

**Michal Dmowski:** Conceptualization, Investigation, Validation, Formal analysis, Visualization, Writing – original draft, Writing – review & editing. **Karolina Makiela-Dzbenska:** Investigation, Validation. **Malgorzata Jedrychowska:** Investigation, Validation. **Milena Denkiewicz-Kruk:** Investigation. **Iwona J. Fijalkowska:** Conceptualization, Validation, Funding acquisition.

## Declaration of Competing Interest

The authors declare that they have no known competing financial interests or personal relationships that could have appeared to influence the work reported in this paper.

## Data Availability

Mutation spectrum data for Saccharomyces cerevisiae psf1-1 pol2-M644G mutants (Original data) (Mendeley Data).
